# Surveillance for avian influenza viruses in wild birds at live bird markets, Egypt, 2014‐2016

**DOI:** 10.1111/irv.12634

**Published:** 2019-02-03

**Authors:** Ahmed S. Kayed, Ahmed Kandeil, Mokhtar R. Gomaa, Rabeh El‐Shesheny, Sara Mahmoud, Nabil Hegazi, Mohamed Fayez, Basma Sheta, Pamela P. McKenzie, Richard J. Webby, Ghazi Kayali, Mohamed A. Ali

**Affiliations:** ^1^ Environmental Research Division, Water Pollution Research Department, Center of Scientific Excellence for Influenza Viruses National Research Centre (NRC) Giza Egypt; ^2^ Department of Infectious Diseases St Jude Children's Research Hospital Memphis Tennessee; ^3^ Faculty of Agriculture, Department of Microbiology Cairo University Giza Egypt; ^4^ Faculty of Science, Zoology Department Damietta University New Damietta Egypt; ^5^ Department of Epidemiology, Human Genetics, and Environmental Sciences University of Texas Houston Texas; ^6^ Human Link Baabda Lebanon

## Abstract

**Aim:**

Egypt is the habitat for a large number of bird species and serves as a vital stopover for millions of migratory birds during their annual migration between the Palearctic and Afrotropical ecozones. Surveillance for avian influenza viruses (AIVs) is critical to assessing risks for potential spreading of these viruses among domestic poultry. Surveillance for AIV among hunted and captured wild birds in Egypt was conducted in order to understand the characteristics of circulating viruses.

**Methods:**

Sampling of wild bird species occurred in two locations along the Mediterranean Coast of Egypt in the period from 2014 to 2016. A total of 1316 samples (cloacal and oropharyngeal swabs) were collected from 20 different species of hunted or captured resident and migratory birds sold at live bird markets. Viruses were propagated then sequenced. Phylogenetic analysis and receptor binding affinities were studied.

**Results:**

Eighteen AIVs (1.37%) were isolated from migratory Anseriformes at live bird markets. Further characterization of the viral isolates identified five hemagglutinin (H3, H5, H7, H9, and H10) and five neuraminidase (N1, N2, N3, N6, and N9) subtypes, which were related to isolates reported in the Eurasian region. Two of the 18 isolates were highly pathogenic H5N1 viruses related to clade 2.2.1, while three isolates were G1‐like H9N2 viruses.

**Conclusions:**

Our data show significant diversity of AIVs in Anserifromes sold at live bird markets in Egypt. This allows for genetic exchanges between imported and enzootic viruses and put the exposed humans at a higher risk of infection.

## 1 INTRODUCTION

Wild birds, particularly waterfowl, are the natural reservoir of many subtypes of influenza A viruses and play an important role in the evolution and spread of avian influenza viruses (AIV).^1^ Birds of the orders *Anseriformes *and *Charadriiformes* are thought to be the most common reservoirs of subtypes H1‐H16 of influenza A viruses.^2^ AIVs are classified into highly pathogenic avian influenza virus (HPAIV) and low pathogenic avian influenza virus (LPAIV). HPAIV H5 Goose/Guangdong lineage can be transmitted to domestic poultry by infected wild birds and spread rapidly, causing serious disease with high mortality.^3^ Other subtypes such as LPAIV H5 and H7 can become highly pathogenic within the domestic species population.^4^ LPAIVs of subtypes H1 to H16 show mild or no disease in wild birds but can infect other hosts sharing their habitat and transmit the virus to highly susceptible poultry species such as chickens, turkeys, and other bird species.^5^ Since several human infections with HPAIVs (eg, H5N1 and H7N7) and LPAIVs (eg, H7N9, H9N2, H10N8)^6^, ^7^ occurred over the last two decades, surveillance has been intensified in both poultry and wild avian life in order to understand disease evolution, virus spread, and risk factors associated with infection.^8^, ^9^, ^10^

Egypt has habitats to a large number of bird species. The wetlands of the northern Nile Delta are a vital stopover for millions of migratory birds during their annual migration between the Palearctic and Afrotropical ecozones.^11^ Two migratory birds flyways, the Black Sea‐Mediterranean and East African‐West Asian flyway, overlap in Egypt.^12^ Therefore, the Egyptian environment is an important site on the wild birds migration network through the old world.^13^

Even though surveillance of wild birds for AIVs has increased substantially worldwide in the last years, few studies have been conducted in Egypt.^14^, ^15^ Here, we conducted active surveillance of AIVs in resident and migratory wild birds either hunted or captured to be sold at live bird markets in Egypt from 2014 to 2016.

## 2 MATERIAL AND METHODS

### 2.1 Collection of specimens

During the period from 2014 to 2016, a total of 1316 samples (658 oropharyngeal swabs and 658 cloacal swabs from 658 birds) were collected from nine resident wild bird species of the orders Gruiformes, Passeriformes, Coraciiformes, Charadriifromes, Strigiformes, and Columbiformes (39 birds) and eleven migratory bird species of the orders Charadriiformes, Passeriformes, Gruiformes, Galliformes, Caprimulgiformes, and Anserifromes (619 birds). Among resident bird species, Passeriformes and Columbiformes were most commonly sampled, 22 and 40 samples, respectively. Among migratory birds, Anseriformes and Galliformes were most commonly sampled, 630 and 482 samples, respectively. Sampling was performed between October and April (72 samples in 2014, 778 samples in 2015, and 466 in 2016). The swabs were collected in transport medium containing 50% glycerol, 50% phosphate‐buffered saline (PBS), penicillin (2×10^6^ U/L), streptomycin (200 mg/L), and amphotericin B (250 mg/L) (Lonza, Walkersville, MD, USA). During the study period, swab samples were collected from two different locations of wetlands along the Egyptian Mediterranean coast: Damietta (northern east Nile Delta; 1238 samples) and El‐Arish (North Sinai; 78 samples). Samples were obtained from hunted migratory quail (Galliformes) from El‐Arish in 2015 only or captured migratory or resident wild birds being sold at live bird markets in Damietta. All samples were collected from apparently healthy birds. The specimens were stored on ice and transported rapidly for laboratory processing.

### 2.2 Virus detection, isolation and subtyping

A volume of 50 mL phosphate‐buffered saline (PBS) containing antibiotic/antimycotic mixture of 10 000 U/mL penicillin, 10 000 μg/mL streptomycin, and 25 μg/mL amphotericin B was used as egg infection medium (Lonza, Walkersville, MD, USA). A volume of 200 μL of the viral inoculum (100 μL sample and 100 μL egg infection medium) was inoculated into the allantoic cavities of 3 10‐day‐old specific pathogen‐free embryonated chicken eggs and incubated for two days at 37°C as per World Health Organization (WHO) guidelines.^16^ The inoculated eggs were tested for influenza virus by hemagglutination assays using 0.5% chicken red blood cell. The positive samples were aliquoted and stored at −80°C.

RNA from each positive sample was extracted using QIAamp Viral RNA Mini Kit (Qiagen, Germany), then typed by M gene using real‐time PCR (RT‐PCR) according to WHO protocol.^17^ The positive M gene samples were further subtyped by RT‐PCR with specific primers for hemagglutinin and neuraminidase genes as previously described.^18^, ^19^

### 2.3 Statistical analysis

Chi‐square and Fisher's exact tests were used to compare positivity rates between sample types (cloacal vs oropharyngeal) and sites (El‐Arish vs Damietta).

### 2.4 Full genome sequencing and analysis of sequences

The first‐strand cDNA was synthesized using Superscript III Reverse transcriptase (Invitrogen, Carlsbad, CA) and Uni‐12 primer (5’AGCRAAAGCAGG3’) as per manufacturer's protocol. Using Phusion Master Mix kit (Thermo Scientific, Wilmington, USA), the desired genes of the isolates from poultry were amplified using universal primers.^20^ Briefly, using gene‐specific primers, 2 µL of each RT‐PCR product was subjected to PCR by an initial denaturation step (98°C for 30 seconds), followed by 40 cycles each consisting of 98°C for 10 seconds, 57°C for 30 seconds, 72°C for 3 minutes, and final elongation (72°C for 10 minutes). Amplicons of the appropriate sizes were subsequently gel purified using QIAGEN gel extraction kit. The purified PCR products were directly used for sequence reactions at Macrogen sequencing facility (Macrogen, South Korea). Sequences were assembled using SeqMan Lasergene 7 software (DNASTAR, Madison, WI, USA). Related sequences for the desired gene were obtained from the Global Initiative on Sharing All Influenza Data (GISAID) (http://platform.gisaid.org/epi3/) in July 2018. Representative sequences of North American and Eurasian lineage strains were obtained from GenBank. Sequence alignments were performed using BioEdit 7.0 software. The phylogenetic tree was constructed using MEGA7 program by applying the neighbor‐joining method with Kimura's two‐parameter substitution model and 1000 bootstrap replicates.

### 2.5 Receptor specificity assay

Virus receptor specificity for six representative AIVs detected in this study was determined as previously described.^21^ 96‐well fetuin‐coated (10 µg/mL) plates were washed with ice‐cold washing buffer (0.01% Tween 80 in 0.23X PBS), blocked with PBS containing 1% bovine serum albumin (BSA), and incubated overnight with 32 hemagglutination units of influenza viruses at 4°C. Plates were washed with washing buffer four times. Biotinylated sialylglycopolymers, 3′‐sialyllactose (SL) (α2,3‐SL, Neu5Acα2‐3Galβ1‐4Glc), and 6′‐sialyllactose (α2,6‐SL, Neu5Acα2‐6Galβ1‐4Glc) (Glycotech, Gaithersburg, MD )were serially diluted in reaction buffer (0.02% Tween 80, 0.02% BSA, 1 µmol/L sialdase inhibitor (Zanamivir), and 1 X PBS) and were added and incubated at 4°C for 2 hours. The plates were washed (4X) and incubated with 100 µL of horseradish peroxidase‐conjugated streptavidin (1:2000) at 4°C for 1 hour. After a final wash, 50 µL of the *o‐*Phenylenediamine (OPD) substrate was added and incubated for 10 minutes at room temperature. The reaction was stopped with 1 N sulfuric acid, and absorbance was measured at 490 nm. Two control viruses, A/Duck/Hong Kong/365/78(H4N6) and A/Hong Kong/1073/99(H9N2), that selectively bind to α2,3‐SL, and α2,6‐SL, respectively, were used.

## 3 RESULTS

### 3.1 Surveillance of AIVs in wild birds

Out of 1316 collected samples from 20 different species of wild birds, 18 samples (1.37%) were positive for AIVs. The viruses were detected in four species of the order Anseriformes captured and sold live at live bird markets in Damietta. The detection rates ranged from 2% to 7%. Northern shoveler had the highest prevalence (6.9%), followed by mallard (5.2%), teal (1.73%), and pintail (1.72%). Of 18 AI isolates, 10 were H7N3 (55.55%), one H7N9 (5.55%), three H9N2 (16.66%), two H5N1 (11.11%), one H10N6 (5.55%), and one H3N6 (5.55%) (Table 1). H5N1 and H9N2 viruses were detected in mallard ducks, while H3N6 was detected in pintails. H10N6 was detected in teal and H7N3 was detected in teal and northern shoveler. No viruses were detected in 2014. In 2015, two H5N1, three H9N2, 2 H7N3, one H10N6, and one H3N6 were detected. Eight H7N3 and one H7N9 were detected in 2016.

**Table 1:** Prevalence of influenza A viruses in samples collected from wild birds in Egypt, 2014‐2016

Variable			Samples collected, positive Samples (%prevalence)	Subtypes (no.)
Sample Type		Oropharyngeal	658, 10(1.52)	H9N2 (2), H7N3 (7), H10N6 (1)
		Cloacal	658, 8(1.21)^a^	H5N1 (2), H9N2 (1), H7N3 (3), H7N9 (1), H3N6 (1)
Species	Common name	Scientific name (order)		
Resident	African swamp hen	*Porphyrio madagascariensis *(Gruiformes)	2, 0(0)	‐
	Moorhen	*Gallinula chloropus *(Gruiformes)	4, 0(0)	‐
	Common chiffchaff	*Phylloscopus collybita *(Passeriformes)	4, 0(0)	‐
	Swallow	*Hirundo rustica rustica *(Passeriformes)	18, 0(0)	‐
	Hooded crow	*Corvus cornix *(Passeriformes)	2, 0(0)	‐
	Common kingfisher	*Alcedo atthis *(Coraciiformes)	4, 0(0)	
	Spur‐winged lapwing	*Vanellus spinosus *(Charadriiformes)	4, 0(0)	‐
	Little owl	*Athene noctua *(Strigiformes)	2, 0(0)	‐
	Laughing dove	*Spilopelia senegalensis *(Columbiformes)	40, 0(0)	‐
Migratory	Common redshank	*Tringa tetanus *(Charadriiformes)	54, 0(0)	‐
	Common greenshank	*Tringa nebularia *(Charadriiformes)	26, 0(0)	‐
	Northern wheatear	*Oenanthe Oenanthe *(Passeriformes)	34, 0(0)	‐
	Common redstart	*Phoenicurus phoenicurus *(Passeriformes)	2, 0(0)	‐
	Coot	*Fulica atra *(Gruiformes)	6, 0(0)	‐
	Common Quail	*Coturnix coturnix *(Galliformes)	482, 0(0)	‐
	Eurasian nightjar	*Caprimulgus europaeus *(Caprimulgiformes)	2, 0(0)	‐
	Mallard	*Anas platyrhynchos *(Anseriformes)	96, 5(5.2)	H5N1 (2), H9N2 (3)
	Northern shoveler	*Anas clypeat *(Anseriformes)	72, 5(6.9)	H7N3 (5)
	Pintail	*Anas acuta *(Anseriformes)	58, 1(1.72)	H3N6 (1)
	Teal	*Anas crecca *(Anseriformes)	404, 7(1.73)	H7N3 (5), H7N9 (1), H10N6 (1)
Site	El‐Arish		78, 0(0)	‐
	Damietta		1238, 18(1.45)^b^	H5N1 (2), H9N2 (3), H7N3 (10), H7N9 (1), H10N6 (1), H3N6 (1)

a Positivity rate comparison between cloacal vs oropharyngeal swabs was not significant by Chi‐square test.

b Positivity rate comparison between sites was not significant by Fisher's exact test.

### 3.2 Phylogenetic analysis

#### 3.2.1 H5N1

**Figure 1:**
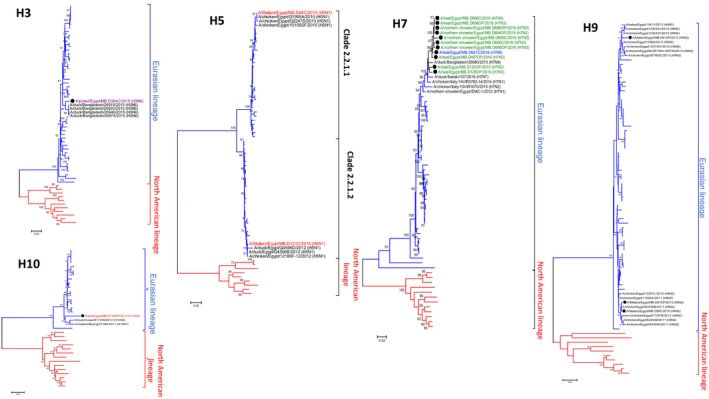
Phylogenetic tree of the nucleotide sequences of different HA subtypes (5, 7, 3, 9, and 10), and NA of AIVs isolated between 2014 and 2016 from wild birds in Egypt. The phylogenetic trees were generated using MEGA version 7. Isolates sequenced specifically for this study are labeled with red squares. The percentage of replicate trees in which the associated taxa clustered together in the bootstrap test (1000 replicates) is shown at the dendrogram nodes

**Figure 2:**
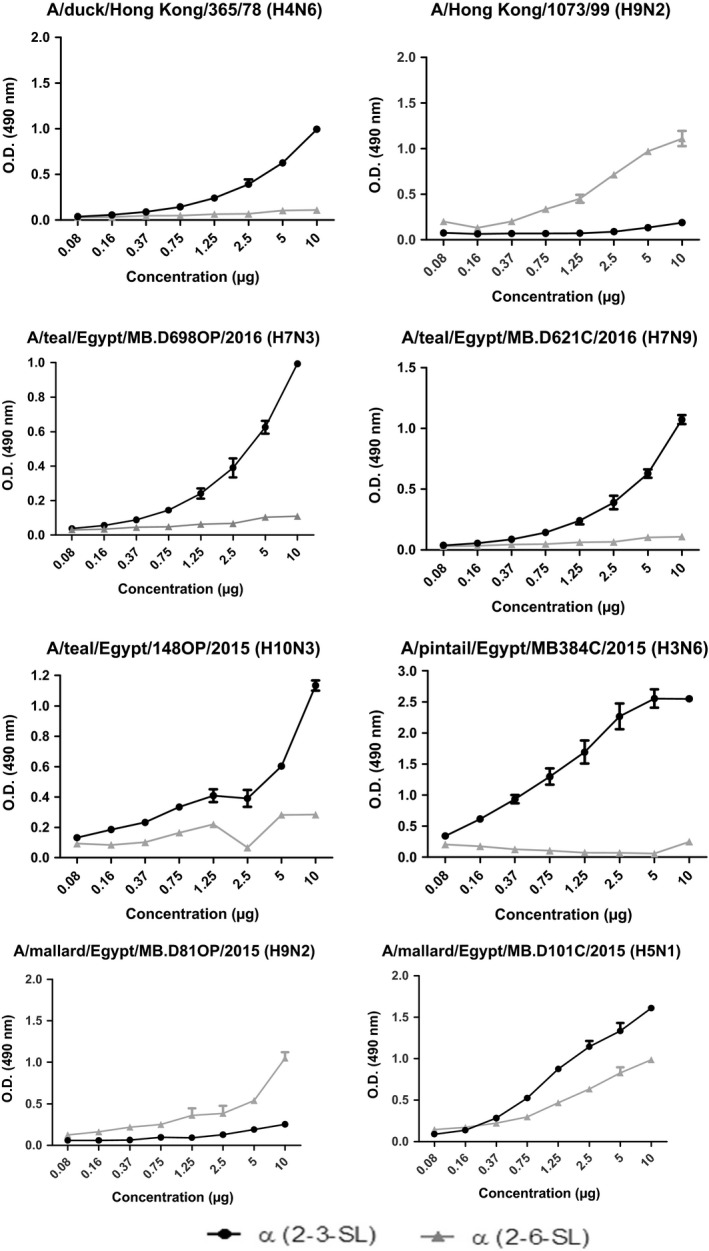
Characterization of the receptor binding affinity of the newly detected H3N6, H5N1, H9N2, H7N3, H7N9, H10N3, AIVs in wild bird in Egypt to two different biotinylated sialylglycopolymers, 3′‐sialyllactose α (2,3‐SL), and 6′‐sialyllactose α (2,6‐SL). H4N6 and H9N2 were used as control viruses with previously established affinities

Phylogenetic analysis for the HA and NA segments of the two H5N1 isolates detected in mallards indicated that A/mallard/Egypt/MB.D84C/2015(H5N1) was closely related to Egyptian isolates of clade 2.2.1.1, while A/mallard/Egypt/MB.D101C/2015(H5N1) was closely related to Egyptian isolates of clade 2.2.1.2 (Figure 1; Figure S1). This was also the case for all the gene segments of these viruses (Figure S2). Both H5N1 viruses had multiple basic amino acids (^321^PQGEGRRKKR/GLF^333^) at the cleavage site of the HA (H5 numbering), which is an indicator of high virulence in chickens as per the Terrestrial Animal Health Code of the World Animal Health Organization (OIE). No clinical signs of HPAI infections (ex. respiratory distress, edema of the head, lethargy, and neurological signs) were detected in the mallard ducks during sampling. An AI A/mallard/Egypt/MB.D101C/2015(H5N1) virus was found to bind preferentially to avian‐like receptor (α2,3‐SL) (Figure 2).

#### 3.2.2 H9N2

All gene segments of the three H9N2 isolates from mallards showed high sequence homology with other Egyptian H9N2 strains that belong to the G1‐like H9N2 viruses (Figure 1; Figures S1 and S2). The HA cleavage site sequence was ^333^PAKSSR*GLF^341^ (according to H9 numbering), which indicates low pathogenicity of H9N2 viruses. A/mallard/Egypt/MB.D 81OP/2015(H9N2) had higher binding preference for human‐like a2,6‐SL receptor than a2,3‐SL avian‐like receptor (Figure 2).

#### 3.2.3 ***H7N3*** ***and H7N9***

We investigated the complete genome of 10 AI H7N3 and one H7N9 viruses. HA cleavage sites of H7 viruses had a motif ^333^PELPKGRGLF^342 ^at the cleavage site, found typically in previously characterized LPAI H7 viruses including human H7N9 viruses detected in China. All isolates had a glutamine at position 235/226 (H7/H3 numbering) and a glycine residue at position 237/228 (H7/H3 numbering), indicating a preferential binding to a2,3‐SL receptors rather than human‐like a2,6‐SL receptor. This was confirmed by the results of the biological receptor binding assay (Figure 2). The HA gene of the detected H7 viruses had a distinguishable cluster related to the Eurasian lineage and was closely related to A/duck/Bangladesh/26980/2015(H7N9). Phylogenetic analyses of the internal genes of the H7N3 viruses revealed dispersion throughout the phylogenetic trees, indicating four genetic constellations forms of H7N3 viruses. A/teal/Egypt/MB.D128OP/2015(H7N3) and A/teal/Egypt/MB.D125OP/2015(H7N3) isolated on the same day at the same site had different genetic constellation forms. This suggests that H7 viruses had undergone extensive reassortment with different Eurasian LPAIVs.

#### 3.2.4 ***H3N6*** ***isolate***

All genes of A/pintail/Egypt/MB.D384C/2015(H3N6) were closely related to Eurasian strains. This isolate had a glutamine at position 226 and a threonine residue at position 228 (H3 numbering). The virus had higher binding preference for α2,3‐SL receptors rather α 2,6‐SL receptors (Figure 2).

#### 3.2.5 ***H10N3*** ***isolate***

Phylogenetic analysis of A/teal/Egypt/MB.D148OP/2015(H10N3) showed that this was closely related to viruses of the Eurasian lineage (Figure 1; Figures S1 and S2). The receptor binding pocket area of this isolate had amino acids 95Y, 151 W, 183H, 190E, 191 K, 194L, 226Q, 227S, 228G, and 229R. None of these amino acid residues have been reported to be involved in the recognition of human‐like receptors,^22^ suggesting that the Egyptian H10N3 isolate likely binds to avian‐like receptors (a2,3‐SL) (Figure 2). The HA cleavage site sequence of the H10N3 isolate was ^330^RGLF^333^ (according to H3 numbering), which is the signature of low pathogenicity of influenza A viruses.

## 4 DISCUSSION

Studying the ecology and evolution of AIVs in wild aquatic birds is key to elucidate their role in virus dissemination among different hosts in different geographic regions. Egypt, where H5 and H9 viruses are enzootic in domestic poultry, acts as a stopover for millions of wild aquatic birds during autumn and spring migration seasons annually providing excellent grounds for reassortment events between AIVs. Several of those species are hunted or captured to be sold live at bird markets. This provides a niche where AIVs can exchange genetic material and where human exposure raises the potential for infection. Therefore, it is important to determine which AIVs are present in wild birds in Egypt and use this information in designing effective control strategies.

The isolated AIVs included five different HA subtypes (3, 5, 7, 9, and 10) and five NA subtypes (1, 2, 3, 6, and 9) indicating that wild birds in Egypt introduce a wide range of viruses into the Egyptian environment. Similar to previous surveillance studies,^1^, ^23^ all isolates were detected in *Anseriformes *(mallard, teal, pintail, and shoveler). These species migrate yearly in the winter season and stop at the Northern coastal area and lakes. According to previous studies, mallards, teals, and northern shovelers constitute important hosts for AIV.^24^ *Anseriformes* play an important role in influenza virus transmission and evolution as they are capable of carrying virus over long distances.^1^, ^25^

H5N1 and H9N2 viruses detected in migratory mallards were shown to possess genes closely related to AIV detected in domestic poultry in Egypt. The sampled wild bird markets had a mix of domestic, wild migratory, and resident wild birds on display to be sold for human consumption. Species shared cages and drinking water; hence, viral transmission could occur. This indicates that wild migrating Anseriformes may acquire infection with enzootic H5 and H9 viruses from domestic birds in the market. However, this evidence comes from captured birds to be sold for human consumption and is not likely to continue their migration. Although minimal, there is a risk of exporting enzootic H5 and H9 AIVs through migratory birds.

Reassortment was observed considerably in all non‐enzootic viruses detected in this study. This is highly evident in the case of H7N3 viruses as was seen in other regions.^26^ The genes of these viruses were related to genes from other Eurasian viruses of various subtypes. This indicates that the migratory bird routes that pass over Egypt are vital in the dissemination of AIVs from Asia into Europe and Africa and that gene‐sharing is occurring somewhere along these routes.

Genetic characterization of the whole genome of ten H7N3 viruses indicated that all eight segments originated from Eurasian wild bird low pathogenic avian influenza. The high heterogeneity of the gene pool found in A/teal/Egypt/MB.D128OP/2015(H7N3) and A/teal/Egypt/MB.D125OP/2015(H7N3) isolated on the same day at the same site is indicative of frequent reassortment events of H7N3 in teal. A previous paper reported the isolation of H7N3, H7N9, H7N1, and H7N7 viruses from wild birds in Egypt.^14^ This indicates that the frequency of isolation of the H7NX viruses in Egypt was high. AI H10N3 and H3N6 were not characterized in previous surveillance reports of AIV in wild birds in Egypt.^14^

Among 4 Anseriformes species infected with AIVs, the northern shoveler (*Anas clypeata*) had the highest prevalence rate as previously shown in a study conducted in other migratory bird flyways.^27^

Based on nucleotide sequences of NS segments, AIVs were classified into alleles A and B.^28^ NS gene of A/Pintail/Egypt/MB.D384C/2015(H3N6) was categorized as allele B with Eurasian LPAIVs, while the remaining isolates were categorized as allele A (Figure S2). Previous studies showed that the allele A AIVs are more common than allele B.^29^, ^30^ Co‐circulation of allele A and B viruses was previously detected in Northern Pintail in Japan during 2007‐2008.^30^

Our findings show the role of wild aquatic birds in transcontinental virus transmission and provide evidence of continuing reassortment of the different subtypes of AIVs.^31^ However, our analysis shows that a major gap in surveillance, especially viral sequences, exists along the flyways that overlap in Egypt. To this effect, most of the sequences that were close to the viruses detected in this study were from Georgia, China, or Bangladesh. This means that our understanding of the evolution and epidemiology of AIVs in the wild birds traveling along Eurasian routes remains minimal. Hence, extensive active surveillance of AIV in wild aquatic birds is essential to enable early warning of newly emerging viruses.

This study may have been affected by several limitations. The sampling scheme we followed was mainly restricted to captured migratory birds being sold at live bird markets in one region in Egypt, Damietta. Although these data enabled us to highlight this setting as a niche for influenza virus transmission, it does not allow us to draw conclusions about the ecology and transmission of AI in wild birds within their natural habitats. The other site we sampled, El‐Arish, was restricted to a single species; hence, comparison to Damietta does not provide a significant conclusion. Furthermore, the isolated viruses were detected after a single 2‐day incubation period in embryonated chicken eggs thus viruses that are not well‐adapted to this host may have been missed hence underestimating the prevalence of AI.

Capturing and selling migratory birds at live bird markets where domestic and resident wild bird species are sold enables the exchange of genetic material between viruses infecting the different species.^32^ Our findings show interspecies transmission as viruses enzootic to poultry were isolated from migratory Anseriformes. On the other hand, the low pathogenic wild bird viruses that were detected may potentially infect domestic species. This makes live bird markets selling non‐domestic bird species an important niche for AIV transmission endangering domestic poultry and elevating the risk of human infection. Considering our findings, it is recommended that biosecurity measures are introduced into these markets and more surveillance is conducted.

## ETHICAL APPROVAL

All applicable international, national, and/or institutional guidelines for the care and use of animals were followed. This article does not contain any studies with human participants performed by any of the authors.

## CONFLICT OF INTEREST

The authors declare no conflict of interest.

## References

[1] Yoon SW , Webby RJ , Webster RG . Evolution and ecology of influenza A viruses. Curr Top Microbiol Immunol. 2014;385:359‐375. 24990620 10.1007/82_2014_396

[2] Alexander DJ . A review of avian influenza in different bird species. Vet Microbiol. 2000;74(1–2):3‐13. 10799774 10.1016/s0378-1135(00)00160-7

[3] Steinhauer DA . Role of hemagglutinin cleavage for the pathogenicity of influenza virus. Virology. 1999;258(1):407‐20. 10.1006/viro.1999.9716 10329563

[4] Dietze K , Graaf A , Homeier‐Bachmann T , et al. From low to high pathogenicity‐Characterization of H7N7 avian influenza viruses in two epidemiologically linked outbreaks. Transbound Emerg Dis. 2018;65(6):1576‐1587. 29790657 10.1111/tbed.12906

[5] Ducatez MF , Webster RG , Webby RJ . Animal influenza epidemiology. Vaccine. 2008;26(Suppl 4):D67‐D69. 19230163 10.1016/j.vaccine.2008.07.064 PMC2735110

[6] Fouchier RA , Schneeberger PM , Rozendaal FW , et al. Avian influenza A virus (H7N7) associated with human conjunctivitis and a fatal case of acute respiratory distress syndrome. Proc Natl Acad Sci USA. 2004;101(5):1356‐1361. 14745020 10.1073/pnas.0308352100 PMC337057

[7] WHO . Cumulative number of confirmed human cases for avian influenza A(H5N1) reported to WHO, 2003–2015. 2015; http://www.who.int/influenza/human_animal_interface/EN_GIP_20151214cumulativeNumberH5N1cases.pdf?ua=1.

[8] Kayali G , Kandeil A , El‐Shesheny R , et al. Avian influenza A(H5N1) virus in Egypt. Emerg Infect Dis. 2016;22(3):379‐388. 26886164 10.3201/eid2203.150593 PMC4766899

[9] Kayali G , Kandeil A , El‐Shesheny R , et al. Active surveillance for avian influenza virus, Egypt, 2010–2012. Emerg Infect Dis. 2014;20(4):542‐551. 24655395 10.3201/eid2004.131295 PMC3966394

[10] Pannwitz G , Wolf C , Harder T . Active surveillance for avian influenza virus infection in wild birds by analysis of avian fecal samples from the environment. J Wildl Dis. 2009;45(2):512‐518. 19395763 10.7589/0090-3558-45.2.512

[11] Denny P . Africa In: Finlayson M, Moser M, eds. Wetlands. London, UK: International Waterfowl and Wetlands Research Bureau; 1991:115‐148.

[12] Si Y , Skidmore AK , Wang T , et al. Spatio‐temporal dynamics of global H5N1 outbreaks match bird migration patterns. Geospat Health. 2009;4(1):65‐78. 19908191 10.4081/gh.2009.211

[13] Alexander DJ . An overview of the epidemiology of avian influenza. Vaccine. 2007;25(30):5637‐5644. 17126960 10.1016/j.vaccine.2006.10.051

[14] Gerloff NA , Jones J , Simpson N , et al. A high diversity of Eurasian lineage low pathogenicity avian influenza A viruses circulate among wild birds sampled in Egypt. PLoS ONE. 2013;8(7):e68522. 23874653 10.1371/journal.pone.0068522 PMC3710070

[15] Saad MD , Ahmed LS , Gamal‐Eldein MA , et al. Possible avian influenza (H5N1) from migratory bird, Egypt. Emerg Infect Dis. 2007;13(7):1120‐1121. 18214200 10.3201/eid1307.061222 PMC2878221

[16] WHO . Manual for the laboratory diagnosis and virological surveillance of influenza. 2011.

[17] WHO . WHO Manual on Animal Influenza Diagnosis and Surveillance. Geneva: WHO; 2002; http://www.wpro.who.int/emerging_diseases/documents/docs/manualonanimalaidiagnosisandsurveillance.pdf?ua=1.

[18] Lee MS , Chang PC , Shien JH , Cheng MC , Shieh HK . Identification and subtyping of avian influenza viruses by reverse transcription‐PCR. J Virol Methods. 2001;97(1–2):13‐22. 11483213 10.1016/s0166-0934(01)00301-9

[19] Fereidouni SR , Starick E , Grund C , et al. Rapid molecular subtyping by reverse transcription polymerase chain reaction of the neuraminidase gene of avian influenza A viruses. Vet Microbiol. 2009;135(3–4):253‐260. 19028027 10.1016/j.vetmic.2008.09.077

[20] Hoffmann E , Stech J , Guan Y , Webster RG , Perez DR . Universal primer set for the full‐length amplification of all influenza A viruses. Arch Virol. 2001;146(12):2275‐2289. 11811679 10.1007/s007050170002

[21] Matrosovich MN , Gambaryan AS . Solid‐phase assays of receptor‐binding specificity. Methods Mol Biol. 2012;865:71‐94. 22528154 10.1007/978-1-61779-621-0_5

[22] Deng G , Shi J , Wang J , et al. Genetics, receptor binding, and virulence in mice of H10N8 influenza viruses isolated from ducks and chickens in live poultry markets in China. J virol. 2015;89(12):6506‐6510. 25855738 10.1128/JVI.00017-15 PMC4474319

[23] Douglas KO , Lavoie MC , Kim LM , Afonso CL , Suarez DL . Isolation and genetic characterization of avian influenza viruses and a Newcastle disease virus from wild birds in Barbados: 2003–2004. Avian Dis. 2007;51(3):781‐787. 17992942 10.1637/0005-2086(2007)51[781:IAGCOA]2.0.CO;2

[24] Wallensten A , Munster VJ , Latorre‐Margalef N , et al. Surveillance of influenza A virus in migratory waterfowl in northern Europe. Emerg Infect Dis. 2007;13(3):404‐411. 17552093 10.3201/eid1303.061130 PMC2725893

[25] Webster RG , Bean WJ , Gorman OT , Chambers TM , Kawaoka Y . Evolution and ecology of influenza A viruses. Microbiol Rev. 1992;56(1):152‐179. 1579108 10.1128/mr.56.1.152-179.1992 PMC372859

[26] Dhingra MS , Artois J , Dellicour S , et al. Geographical and historical patterns in the emergences of novel highly pathogenic avian influenza (HPAI) H5 and H7 viruses in poultry. Front Vet Sci. 2018;5:84. 29922681 10.3389/fvets.2018.00084 PMC5996087

[27] Hill NJ , Takekawa JY , Cardona CJ , et al. Cross‐seasonal patterns of avian influenza virus in breeding and wintering migratory birds: a flyway perspective. Vector Borne Zoonotic Dis. 2012;12(3):243‐253. 21995264 10.1089/vbz.2010.0246 PMC3300065

[28] Treanor JJ , Snyder MH , London WT , Murphy BR . The B allele of the NS gene of avian influenza viruses, but not the A allele, attenuates a human influenza A virus for squirrel monkeys. Virology. 1989;171(1):407‐9. 10.1016/0042-6822(89)90504-7 2525836

[29] Zohari S , Gyarmati P , Ejdersund A , et al. Phylogenetic analysis of the non‐structural (NS) gene of influenza A viruses isolated from mallards in Northern Europe in 2005. Virol J. 2008;5:147. 19077274 10.1186/1743-422X-5-147 PMC2625346

[30] Jahangir A , Ruenphet S , Sultana N , Shoham D , Takehara K . Genetic analysis of avian influenza viruses: cocirculation of avian influenza viruses with allele A and B nonstructural gene in northern pintail (Anas acuta) ducks wintering in Japan. Influenza Res Treat. 2012;2012:847505. 23320157 10.1155/2012/847505 PMC3540751

[31] Ramey AM , Pearce JM , Ely CR , et al. Transmission and reassortment of avian influenza viruses at the Asian‐North American interface. Virology. 2010;406(2):352‐359. 20709346 10.1016/j.virol.2010.07.031

[32] Hassan MM , Hoque MA , Ujvari B , Klaassen M . Live bird markets in Bangladesh as a potentially important source for Avian Influenza Virus transmission. Prev Vet Med. 2018;156:22‐27. 29891142 10.1016/j.prevetmed.2018.05.003

