# Supplementary material for: Surveillance for avian influenza viruses in wild birds at live bird markets, Egypt, 2014‐2016
Source: Influenza Other Respir Viruses. 2019 Feb 3;13(4):407–14. doi: 10.1111/irv.12634 (PMC6586179; doi:10.1111/irv.12634)
Supplement: Supplementary file 1 [file IRV-13-407-s001.pptx]

## Slide 1
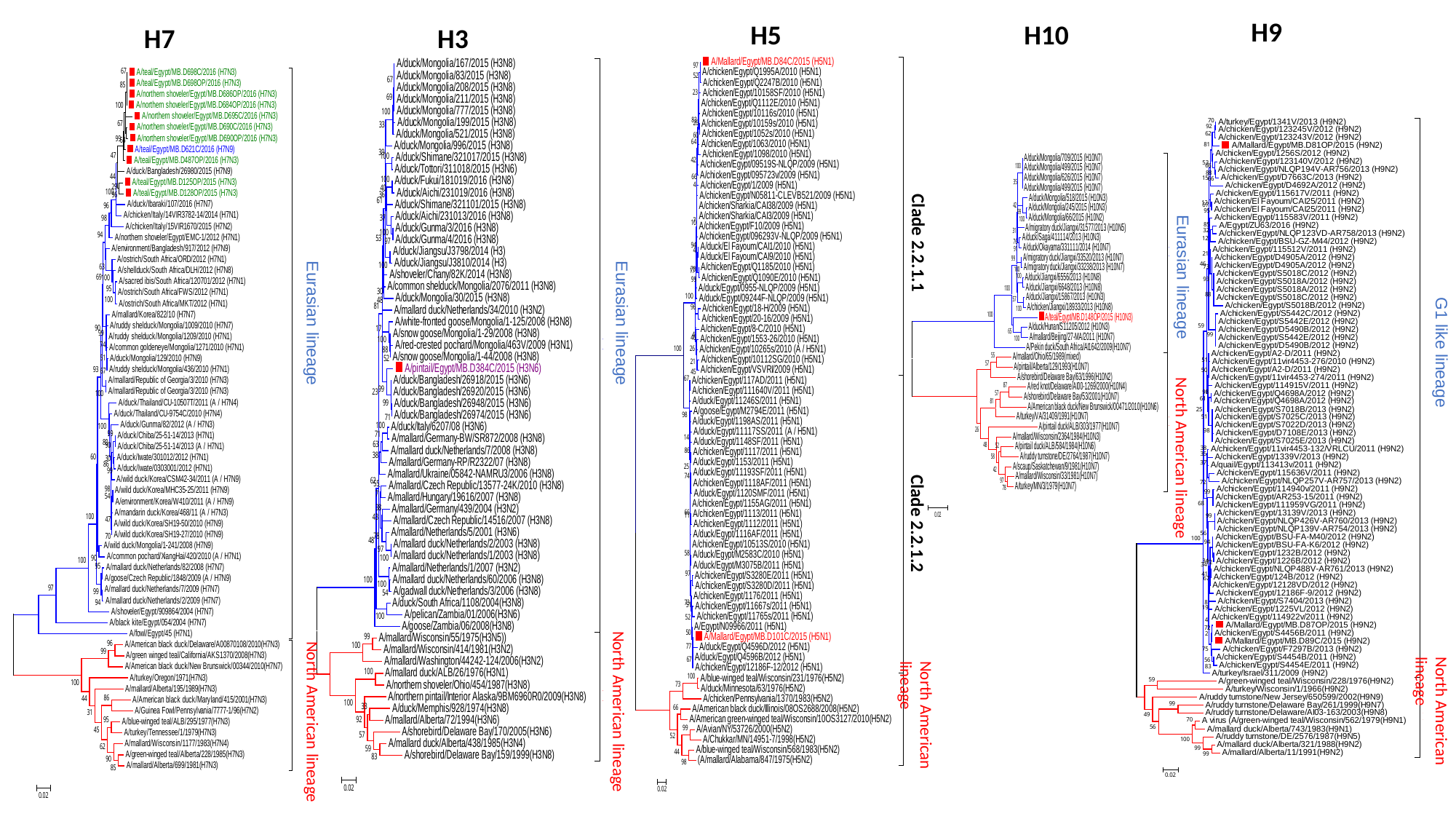

H9
H5
H10
H7
H3
Eurasian lineage
Clade 2.2.1.1
Eurasian lineage
Eurasian lineage
G1 like lineage
North American lineage
Clade 2.2.1.2
North American lineage
North American lineage
North American lineage
North American lineage
