# Supplementary material for: Surveillance for avian influenza viruses in wild birds at live bird markets, Egypt, 2014‐2016
Source: Influenza Other Respir Viruses. 2019 Feb 3;13(4):407–14. doi: 10.1111/irv.12634 (PMC6586179; doi:10.1111/irv.12634)
Supplement: Supplementary file 2 [file IRV-13-407-s002.docx]

**PB2**

**PB1**

**PA**

**NP**

**M**

**NS**

**NS allele A**

**.0**

**NS allele B**

**.0**
